# Temperature impacts on fish physiology and resource abundance lead to faster growth but smaller fish sizes and yields under warming

**DOI:** 10.1111/gcb.16341

**Published:** 2022-08-11

**Authors:** Max Lindmark, Asta Audzijonyte, Julia L. Blanchard, Anna Gårdmark

**Affiliations:** ^1^ Department of Aquatic Resources, Institute of Coastal Research Swedish University of Agricultural Sciences Öregrund Sweden; ^2^ Nature Research Centre Vilnius Lithuania; ^3^ Institute for Marine and Antarctic Studies and Centre for Marine Socioecology University of Tasmania Hobart Tasmania Australia; ^4^ Department of Aquatic Resources Swedish University of Agricultural Sciences Uppsala Sweden

**Keywords:** body size, climate change, fisheries yield, food web, metabolic theory, multi species, size spectrum

## Abstract

Resolving the combined effect of climate warming and exploitation in a food web context is key for predicting future biomass production, size‐structure and potential yields of marine fishes. Previous studies based on mechanistic size‐based food web models have found that bottom‐up processes are important drivers of size‐structure and fisheries yield in changing climates. However, we know less about the joint effects of ‘bottom‐up’ and physiological effects of temperature; how do temperature effects propagate from individual‐level physiology through food webs and alter the size‐structure of exploited species in a community? Here, we assess how a species‐resolved size‐based food web is affected by warming through both these pathways and by exploitation. We parameterize a dynamic size spectrum food web model inspired by the offshore Baltic Sea food web, and investigate how individual growth rates, size‐structure, and relative abundances of species and yields are affected by warming. The magnitude of warming is based on projections by the regional coupled model system RCA4‐NEMO and the RCP 8.5 emission scenario, and we evaluate different scenarios of temperature dependence on fish physiology and resource productivity. When accounting for temperature‐effects on physiology in addition to on basal productivity, projected size‐at‐age in 2050 increases on average for all fish species, mainly for young fish, compared to scenarios without warming. In contrast, size‐at‐age decreases when temperature affects resource dynamics only, and the decline is largest for young fish. Faster growth rates due to warming, however, do not always translate to larger yields, as lower resource carrying capacities with increasing temperature tend to result in decline in the abundance of larger fish and hence spawning stock biomass. These results suggest that to understand how global warming affects the size structure of fish communities, both direct metabolic effects and indirect effects of temperature via basal resources must be accounted for.

## Graphical abstract

In this study, we calibrated a temperature dependent multi‐species size spectrum model to the Baltic Sea offshore community. We project it at different fishing rates and temperatures, and evaluate how individual growth rates, population size structure and fisheries yields are affected. We find that warming generally increases growth rates of fishes, but the population size structure shifts towards smaller bodied individuals. These results lead to smaller fisheries yields, as less biomass is found in the larger size classes, but maximum sustainable fishing rates occur at higher levels because growth rates are higher.

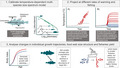

## 1 INTRODUCTION

Climate change affects aquatic food webs directly by affecting species' distribution (Pinsky et al., 2013), abundance (McCauley et al., 2015), body size (Baudron et al., 2014; Daufresne et al., 2009), and ecosystem function (Pontavice et al., 2019). Global retrospective analysis of warming and fish population dynamics has revealed that the maximum sustainable yield of scientifically assessed fish populations across ecoregions has already declined by 4.1% on average between 1930 and 2010 due to climate change (Free et al., 2019). These results are also matched in magnitude and direction by projections from an ensemble of mechanistic ecosystem models, which predict ~5% decline in animal biomass for every 1°C of warming, especially at higher trophic levels (Lotze et al., 2019). Across a range of process‐based ecosystem models, declines in the productivity of fish stocks and abundance of large fish have been linked to changes in primary production or zooplankton abundance (Barange et al., 2014; Blanchard et al., 2012; Heneghan et al., 2021; Lotze et al., 2019; Tittensor et al., 2021; Woodworth‐Jefcoats et al., 2013, 2015). However, even in areas where warming is predicted to have positive effects on primary production, fish productivity does not appear to increase (Free et al., 2019). This suggests that fish population dynamics might be strongly influenced by other factors, such as temperature‐driven changes in recruitment, mortality or somatic growth (Free et al., 2019), yet the driving mechanisms remain poorly understood.

Global warming is also predicted to cause reductions in the adult body size of organisms, and this is often referred to as the third universal response to warming (Daufresne et al., 2009; Forster et al., 2012; Sheridan & Bickford, 2011). It is often attributed to the temperature‐size rule (TSR), which is observed in a wide range of ectotherms (Forster et al., 2012). This is an intraspecific rule stating that individuals reared at warmer temperatures develop faster, mature earlier but reach smaller adult body sizes (Atkinson, 1994; Ohlberger, 2013). In line with TSR expectations, faster growth rates or larger size‐at‐age of young life stages are commonly found in both experimental, field data and modelling studies (Baudron et al., 2014; Huss et al., 2019; Neuheimer et al., 2011; Neuheimer & Grønkjaer, 2012; Ohlberger et al., 2011; Thresher et al., 2007; van Dorst et al., 2019). Similarly, declines in maximum or asymptotic body size of fish have been reported to correlate with warming trends for a number of commercially exploited marine fishes (Baudron et al., 2014; Ikpewe et al., 2020; van Rijn et al., 2017). However, in intensively fished stocks, observed adult body sizes can decrease also for other reasons, including direct removals of large fish, or evolution towards earlier maturing and fast growth in response to fishing (Audzijonyte et al., 2013; Jørgensen et al., 2007). Moreover, decreasing adult fish size in warming waters is by far not universal. For example, no clear negative effects of warming on the body size or growth of large fish could be found in a recent experimental study (Barneche et al., 2019), or in a semi‐controlled lake heating experiment (Huss et al., 2019). Similarly, across 335 coastal fish species mean species body size was similarly likely to be larger or smaller in warmer waters (Audzijonyte et al., 2020). Also, Tu et al. (2018) found that temperature had a relatively minor effect on fish size structure compared to fishing, when assessing 28 stocks from the North Sea, US west coast and Eastern Bering Sea. Even when combined with fishing, only 44% of variation in size structure could be explained. Thus, the effects of temperature on body sizes may be more complex than often depicted, and we still do not fully understand the mechanisms by which temperature affects growth and body size over ontogeny (Audzijonyte et al., 2019; Ohlberger, 2013). Increasing our understanding of these mechanisms is important because body size is a key trait in aquatic ecosystems (Andersen et al., 2016) and warming‐induced changes in growth and size‐at‐age of fish populations could have implications not only for biomass and productivity, but also ecosystem structure and stability (Audzijonyte et al., 2013).

Physiologically structured models can address the complex interplay of direct and indirect temperature impacts on food webs, as they account for the food and size dependence of body growth through ecological interactions using bioenergetic principles. Recent applications have demonstrated decreasing maximum body sizes in fish communities due to changes in plankton abundance or size (Woodworth‐Jefcoats et al., 2019). Similar body size responses emerge in models that focus on temperature‐dependence of physiological processes, such as metabolism and feeding rates (Guiet et al., 2016; Lefort et al., 2015; Woodworth‐Jefcoats et al., 2019), but to what extent these community body size shifts are driven by declining abundance of large fish versus changes in size‐at‐age across a range of ages remains unclear.

To explore how direct and indirect effects of warming impact marine food web size structure and fisheries yields, we evaluate the impacts of temperature‐driven changes in resource productivity and individual fish physiology using an example case of the Baltic Sea. The Baltic Sea constitutes a great example system, as it is a relatively well understood and species poor system (Casini et al., 2009; Mackenzie et al., 2007) that also is one of the warming hotspots globally (Belkin, 2009). Using a temperature‐dependent size spectrum model, we analyse a set of different scenarios where either fish physiology, basal resources, or both depend on temperature, and contrast these scenarios to one another and to non‐warming scenarios. We investigate the mechanisms of warming effects on body growth trajectories (size‐at‐age), average body sizes, population size‐structure and fisheries yields and reference points.

## 2 MATERIALS AND METHODS

In this section, we will describe the food web the model is parameterized to, the equations of the multi‐species size spectrum model, how temperature dependence is implemented in the model, how the model is calibrated and lastly, how the effects of temperature are evaluated.

### 2.1 Food web

**FIGURE 1:**
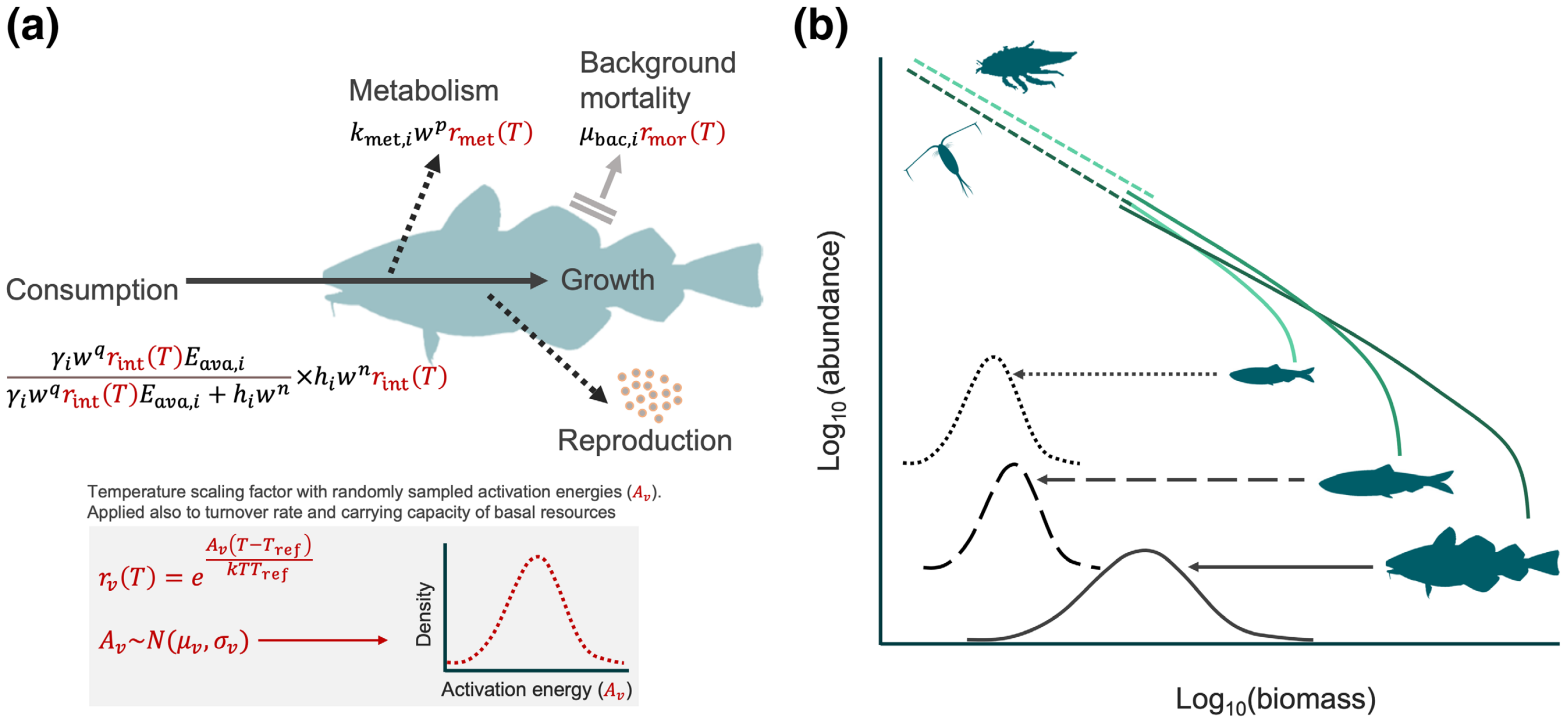
(a) Schematic representation of the individual level energy fluxes and their temperature dependence and (b) the abundance spectrum of fishes (solid lines) emerging from food‐dependent growth and mortality, and spectra of their background pelagic and benthic resource (dashed lines).

We developed a multi‐species size spectrum model (MSSM; Scott et al., 2014), parameterized to represent a simplified version of the food web in the offshore pelagic south‐central Baltic Sea ecosystem (Baltic proper; ICES sub divisions 25–29 + 32, Figure S2) and account for temperature‐dependence of processes within and between individuals (Figure 1). This size structured food web is characterized here by three fish species: Atlantic cod (*Gadus morhua*), sprat (*Sprattus sprattus*) and herring (*Clupea harengus*), and two background resource spectra constituting food for small fish (pelagic and benthic resources). In the south‐central Baltic Sea, these fish species are dominant in terms of biomass, they are the most important species commercially and they all have analytical stock assessments (ICES, 2021). The pelagic background resource spectrum represents mainly phyto‐ and zooplankton while the benthic background resource spectrum represents benthic invertebrates, gobiidaes and small flatfish.

### 2.2 Size spectrum model

The model is based on source code for the multi‐species implementation of size spectrum models in the ‘R'‐package *mizer* (v1.1; Blanchard et al., 2014; R Core Team, 2020; Scott et al., 2014), which has been extended to include multiple background resources (https://github.com/sizespectrum/mizerMR) and temperature‐scaling of key physiological processes. In this section, we describe the key elements of the MSSM using the same notation when possible as in previous multispecies *mizer* models for consistency (Blanchard et al., 2014; Scott et al., 2014).

In MSSMs, individuals are characterized by their weight (w) and species identity (i). The core equation is the McKendrik‐von Foerster equation. Here, it describes the change in abundance‐at‐size through time, $N_{i}\left(w\right)$, from food dependent somatic growth and mortality, based on bioenergetic principles (explicit modelling of energy acquisition and use):

(1) $$\frac{\partial N_{i}\left(w\right)}{\partial t}+\frac{\partial g_{i}\left(w\right)N_{i}\left(w\right)}{\partial w}=-\mu_{i}\left(w\right)N_{i}\left(w\right),$$

where $g_{i}\left(w\right)$ ( $g\;{\text{year}}^{-1}$ ) is somatic growth (dependent on the availability of food) and $\mu_{i}\left(w\right)\left({\text{year}}^{-1}\right)$ is total mortality. At the boundary weight ($w_{0}$, egg size), the influx of individuals is given by constant recruitment, which occurs at every model time step. Total mortality is the sum of the background, starvation, fishing and predation mortality. The constant species‐specific allometric background mortality, $\mu_{\mathrm{bac},i}$, representing density and predation independent sources of mortality, such as ageing, diseases, predation from species not included in the model, depends on the asymptotic weight of a species $W_{i}^{n-1}$ and is given by:

(2) $$\begin{array}{c} \mu_{\mathrm{bac},i}=\mu_{0}W_{i}^{n-1}, \end{array}$$

where n is the mass‐exponent of maximum consumption rate (Hartvig et al., 2011) and $\mu_{0}$ is an allometric constant. Starvation mortality ($\mu_{\mathrm{stv},i}$) is assumed to be proportional to energy deficiency (defined in Equation 11) and inversely proportional to body mass (weight, w), and is defined as

(3) $$\begin{array}{c} \mu_{\mathrm{stv},i}\left(w\right)=\left\{\begin{array}{cc} 0 & \alpha f_{i}\left(w\right)h_{i}w^{n}>k_{\mathrm{met},i}w^{p}\\ \frac{k_{\mathrm{met},i}w^{p}-\alpha f_{i}\left(w\right)h_{i}w^{n}}{\mathrm{\xi w}} & \text{otherwise} \end{array}\right., \end{array}$$

where ξ, the fraction of energy reserves, is 0.1 (Hartvig et al., 2011). Instantaneous fishing mortality ($\mu_{\mathrm{fis},i}$) ($\mathrm{yea}r^{-1}$) is defined as

(4) $$\begin{array}{c} \mu_{\mathrm{fis},i}\left(w_{i}\right)=S_{i}\left(w\right)F_{i}, \end{array}$$

where $S_{i}$ is the selectivity (for simplicity, we assumed knife‐edge selectivity with weight at first catch corresponding to weight at maturation), and $F_{i}$ is fishing mortality. Predation mortality $\left(\mu_{\mathrm{pre},p}\right)$ for prey p (resource or species) equals the amount consumed by predator species i with weight $w_{i}$ :

(5) $$\begin{array}{c} \mu_{\mathrm{pre},p}\left(w_{p}\right)=\sum_{i}\theta_{i,p}\int \phi_{i}\left(\frac{w_{p}}{w_{i}}\right)\left(1-f_{i}\left(w\right)\right)\gamma_{i}w^{q}N_{i}\left(w\right)\mathrm{dw}, \end{array}$$

where $\theta_{i,p}$ is the non size‐based preference of species i on prey p, and $\phi_{i}$ describes the weight‐based preference from the log‐normal selection model (see below; Ursin, 1973). Satiation determines the feeding level $f_{i}\left(w\right)$ and is represented in the model with a Holling functional response type II. Satiation varies from 0 (no satiation) to 1 (full satiation):

(6) $$\begin{array}{c} f_{i}\left(w\right)=\frac{E_{\mathrm{enc},i}\left(w\right)}{E_{\mathrm{enc},i}\left(w\right)+h_{i}w^{n}}, \end{array}$$

where $h_{i}w^{n}$ is the allometric maximum consumption rate and $E_{\mathrm{enc},i}\left(w\right)$ is the encountered food (mass per time). The amount of encountered food for a predator of body weight w is given by the available food in the system multiplied with the search volume, $\gamma_{i}w^{q}$. Here, available food, $E_{\mathrm{ava},i}$, is the integral of the biomass of prey (below split by resource R and species j) that falls within the prey preference ($\theta_{i,p}$) and size‐selectivity ($\phi_{i}$) of predator species i:

(7) $$\begin{array}{c} E_{\mathrm{ava},i}\left(w\right)=\int \left(\sum_{R}\theta_{i,R}N_{R}\left(w_{R}\right)+\sum_{j}\theta_{i,j}N_{j}\left(w_{j}\right)\right)\phi_{i}\left(\frac{w_{p}}{w_{i}}\right)w_{p}dw_{p}, \end{array}$$

where $\theta_{i,R}$ is the preference of species i for resource R, and $\theta_{i,j}$ is the preference of species i on species j. Note that in contrast to other MSSMs (e.g., Blanchard et al., 2014) species in our model do not have specific preferences for other size‐structured species (all values in the species interaction matrix $\theta_{i,j}$ are set to 1). However, they have different preferences for the two background resources. This helps to account for different feeding of sprat, herring and cod on benthic and pelagic resources. The size‐selectivity of feeding, $\phi_{i}\left(\frac{w_{p}}{w_{i}}\right)$, is given by a log‐normal selection function (Ursin, 1967):

(8) $$\begin{array}{c} \phi_{i}\left(\frac{w_{p}}{w_{i}}\right)=\exp\;\left[\frac{-{\left(\ln\left(\frac{w_{i}}{\left(w_{p}\beta_{i}\right)}\right)\right)}^{2}}{2\sigma_{i}^{2}}\right], \end{array}$$

where parameters $\beta_{i}$ and $\sigma_{i}$ are the preferred predator–prey mass ratio and the standard deviation of the log‐normal distribution, respectively. The amount of available prey of suitable sizes (Equation 7) is multiplied with the allometric function describing the search volume ($\gamma_{i}w^{q}$) to get the food actually encountered. The allometric search volume coefficient is calculated as

(9) $$\begin{array}{c} \gamma_{i}\left(f_{0}\right)=\frac{f_{0}h_{i}\beta_{i}^{2-\lambda }\exp\left(-\frac{{\left(\lambda -2\right)}^{2}\sigma_{i}^{2}}{2}\right)}{\left(1-f_{0}\right)\sqrt{2\pi }\kappa \sigma_{i}} \end{array}$$

(Andersen & Beyer, 2006; Scott et al., 2014). Hence the actual biomass of food encountered, $E_{\mathrm{enc},i}\left(w\right)$, is defined as

(10) $$\begin{array}{c} E_{\mathrm{enc},i}\left(w\right)=\gamma_{i}w^{q}E_{\mathrm{ava},i}\left(w\right), \end{array}$$

where q is the size‐scaling exponent of the search volume. The rate at which food is consumed is given by the product $f_{i}\left(w\right)h_{i}w^{n}$, which is assimilated with efficiency α and used to cover metabolic costs. Metabolic costs scale allometrically as $k_{\mathrm{met},i}w^{p}$. The net energy, $E_{\mathrm{net},i}$, is thus

(11) $$\begin{array}{c} E_{\mathrm{net}.i}\left(w\right)=\max\left(0,\alpha f_{i}\left(w\right)h_{i}w^{n}-k_{\mathrm{met},i}w^{p}\right), \end{array}$$

which is allocated to growth or reproduction. The allocation to reproduction ($\psi_{i}$) increases smoothly from 0 around the weight maturation, $w_{\mathrm{mat},i}$, to 1 at the asymptotic weight, $W_{i}$, according to the function

(12) $$\begin{array}{c} \psi_{i}={\left[1+{\left(\frac{w}{w_{\mathrm{mat},i}}\right)}^{-m}\right]}^{-1}{\left(\frac{w}{W_{i}}\right)}^{1-n}, \end{array}$$

where m determines the steepness of the energy allocation curve, or how fast the allocation switches from growth to reproduction at around the maturation size (Andersen, 2019). This function results in the growth rate $g_{i}\left(w\right)$:

(13) $$\begin{array}{c} g_{i}\left(w\right)=E_{\mathrm{net},i}\left(w\right)\left(1-\psi_{i}\left(w\right)\right), \end{array}$$

which approximates a von Bertalanffy growth curve when the feeding level is constant (Andersen, 2019; Hartvig et al., 2011). Reproduction is given by the total egg production in numbers, which is the integral of the energy allocated to reproduction multiplied by a reproduction efficiency factor (ϵ,erepro) divided by the egg weight, $w_{0}$, and the factor 2, assuming only females reproduce

(14) $$\begin{array}{c} R_{\mathrm{phy},i}=\frac{\epsilon }{2w_{0}}\int N_{i\left(w\right)}E_{\mathrm{net},i}\left(w\right)\psi_{i}\left(w\right)\mathrm{dw}. \end{array}$$

This total egg production (or physiological recruitment, $R_{\mathrm{phy},i}$) results in recruits via a Beverton‐Holt stock recruit relationship, such that recruitment approaches a maximum recruitment for a species i ($R_{\max,i}$), as the egg production increases,

(15) $$\begin{array}{c} R_{i}=R_{\max,i}\frac{R_{\mathrm{phy},i}}{R_{\mathrm{phy},i}+R_{\max,i}}, \end{array}$$

where $R_{\max,i}$ is treated as a free parameter and is estimated in the calibration process by minimizing the residual sum of squares between spawning stock biomass from stock assessments and the MSSM. The calibration also ensures that the species coexist in the model.

The temporal dynamics of the background resource ($N_{R}$) spectra (benthic and pelagic) are defined as

(16) $$\begin{array}{c} \frac{\partial N_{R}\left(w,t\right)}{\partial t}=r_{o}w^{p-1}\left[\kappa w^{-\lambda }\left(w\right)-N_{R}\left(w,t\right)\right]-\mu_{\mathrm{pre},R}\left(w\right)N_{R}\left(w,t\right), \end{array}$$

where $r_{o}w^{p-1}$ is the population regeneration rate, $\kappa w^{-\lambda }$ is the carrying capacity of the background resource and $\mu_{\mathrm{pre},R}$ is predation mortality on resource spectrum R and *λ* is defined as 2−n+q (Andersen, 2019).

### 2.3 Temperature dependence of fish physiology and resource dynamics

To study the effects of warming on the modelled ecosystem, we introduce temperature impacts on size‐structured species physiological rates and background resource growth dynamics following the metabolic theory of ecology (MTE; Brown et al., 2004; Gillooly et al., 2001). Temperature affects the rate of metabolism (Clarke & Johnston, 1999; Gillooly et al., 2001), and thus also other biological rates such as feeding and mortality (Brown et al., 2004; Englund et al., 2011; Rall et al., 2012; Thorson et al., 2017). We therefore scale rates of individual metabolism ($k_{\mathrm{met}.i}w^{p}$), maximum consumption ($h_{i}w^{n}$), search volume ($\gamma_{i}w^{q}$) and background mortality ($\mu_{0}W_{i}^{n-1}$) with temperature. Metabolism and consumption are key terms in the energy budget of fish (Equations (11), (12), (13)). Thus, the growth rate is not temperature‐dependent directly but its relationship to temperature emerges from the temperature‐scaling of metabolism and consumption. In *mizer*, metabolism represents all metabolic costs, that is, standard, activity and movement. Henceforth, we assume $k_{\mathrm{met}.i}w^{p}$ scales as standard metabolic rate and refer to it as metabolism or metabolic rate. For simplicity reasons and to reduce the number of parameters and scenarios we assume that all rates scale with temperature exponentially according the Arrhenius temperature correction factor:

(17) $$\begin{array}{c} r\left(T\right)=e^{\frac{A_{v}\left(T-T_{\mathrm{ref}}\right)}{\mathrm{kT}T_{\mathrm{ref}}}}, \end{array}$$

where $A_{v}$ is the activation energy (eV) for individual rate v, T is temperature (K), $T_{\mathrm{ref}}$ is the reference temperature (here 283.15 K, the temperature where the Arrhenius correction factor equals 1) and k is Boltzmann's constant in $\mathrm{eV}\;K^{-1}$ ($=8.617\times 10^{-5}\;\mathrm{eV}\;K^{-1}$). We chose an exponential temperature dependence as it provides a good statistical fit to data, is widely adopted, and because we assume that the projected change in ocean temperature in the studied time range does not lead to temperatures above physiological optima (e.g. Righton et al., 2010 as an example for cod), whereafter physiological rates might be expected to decline. While temperature likely affects other physiological processes as well (such as cost of growth [Barneche et al., 2019] or food conversion efficiency [Handeland et al., 2008]), we focus on the temperature effects on metabolism, maximum consumption, search volume and mortality, as their temperature dependencies are relatively well documented (Brown et al., 2004; Dell et al., 2011; Englund et al., 2011; Lindmark et al., 2022; Pauly, 1980; Thorson et al., 2017).

Temperature also affects plankton and benthos organisms, represented in our model through background resources. In most size spectrum models to date, climate affects primary production (and in some cases zooplankton), and this is modelled by forcing the background spectra to observed abundance‐at‐size of plankton from either remotely sensed variables such as chlorophyll‐*a*, or from output (e.g., net primary production) from earth‐system models (Barange et al., 2014; Blanchard et al., 2012; Canales et al., 2016; Galbraith et al., 2017; Jennings & Collingridge, 2015; Reum et al., 2019; Woodworth‐Jefcoats et al., 2019). These differences have been highlighted as a key source of ecosystem model uncertainties observed in global applications of size‐structured models (Heneghan et al., 2021; Lotze et al., 2019). In order to isolate the effects of temperature on resource and physiological processes, we apply the temperature scaling to the terms of the background resource's semi‐chemostat growth equation (Equation 16), that is, their biomass regeneration rate and carrying capacity. We use the same Arrhenius correction factor with activation energy $A_{r}$, where r refers to background resource parameter. We assume that as temperature goes up, the carrying capacity $\left(\kappa w^{\lambda }\right)$ declines at the same rate as population regeneration $\left(r_{0}w^{p-1}\right)$ rate increases (Gilbert et al., 2014; Savage et al., 2004), that is κ scales with temperature in proportion to $e^{\frac{-A_{r}\left(T-T_{\mathrm{ref}}\right)}{\mathrm{kT}T_{\mathrm{ref}}}}$. This is based on the MTE, which predicts that if nutrient levels are constant, higher respiration rates lead to lower biomasses at carrying capacity (Bernhardt et al., 2018; Savage et al., 2004). To simplify the analyses, our implementation of temperature effects on the background spectrum assumes that its size structure is not affected by the temperature (the slope of the spectrum does not change)—only the overall level of background resources. As an example, using the average activation energy for resource carrying capacity (see next paragraph), the elevation of our background resource spectra (abundance at the geometric mean weight), declines with 8.7% with a 1°C increase in temperature, which is line with a previous study (Heneghan et al., 2019).

Activation energies are estimated with uncertainty and they vary substantially between processes, species, and taxonomic groups. To account for this uncertainty, here we parameterized 200 projections of the food web model using randomly sampled activation energies from normal distributions with rate‐specific means and standard deviations. For metabolism and maximum consumption, we acquired means and standard deviations of posterior distributions provided in the work of Lindmark et al. (2022). For search volume, we assumed that it scales identically to maximum consumption, because both rates are related to feeding processes. For background mortality, we assumed identical scaling to metabolism, as longevity is linked to life span and metabolic rate (Brown et al., 2004; McCoy & Gillooly, 2008; Munch & Salinas, 2009). For background resource activation energies, we use the point estimate of the activation energy (slope from a linear regression of natural log of growth rate as a function of Arrhenius temperature (1/kT) from experimental data in Savage et al. (2004) as the mean. These data consisted of protists, algae and zooplankton, and were extracted using the software WebPlotDigitizer v. 4.1 (Rohatgi, 2012). The standard deviation was approximated by finding the value that resulted in 95% of the normal distribution being within the confidence interval of the linear regression. For each of the 200 parameter combinations, each of the six rate activation energy parameters was sampled independently from their respective distribution and the model was projected to 2050. We then quantified the overall mean response and the ranges of predictions resulting from 200 randomly parameterized simulations and visualized it for the analysis of growth (size‐at‐age) and mean size.

We acknowledge that these scenarios are very simplified for evaluating changes in resource productivity versus physiology with warming, and that they do not necessarily reflect the predicted conditions in the Baltic Sea, nor all the potential pathways by which climate changes affects the environmental conditions in the Baltic Sea. However, the simplicity allows us to contrast effects of warming on basal food resources versus individual physiology of fish.

### 2.4 Model calibration

Here, we present a summary of the calibration approach—a more detailed description of the step‐by‐step calibration protocol can be found in *Model calibration and validation*, Appendix S1. The model was calibrated to average spawning stock biomasses ($\mathrm{SS}B_{i}$) from stock assessment data for cod, herring and sprat (ICES, 2013, 2015) in 1992–2002, using average fishing mortalities ($F_{i}$) in the same time frame. Ideally, the period for calibration should exhibit relative stability, but such periods do not exist in the Baltic Sea, which is greatly influenced by anthropogenic activities and has undergone dramatic structural changes over the last four decades (Möllmann et al., 2009). We chose to calibrate our model to the time period of 1992–2002 as in Jacobsen et al. (2017), which is a period after an ecological regime shift, characterized by high fishing mortality on cod, low cod and herring abundance and high sprat abundance (Gårdmark et al., 2015; Figure S4). The cut‐off at 2002 also ensured that we did not calibrate the model to the period starting from mid 2000's when the growth capacity, condition, proportion of large fish in the population and reproductive capacity of cod started to decline rapidly (Casini et al., 2016; Mion et al., 2018, 2021; Neuenfeldt et al., 2020; Svedäng & Hornborg, 2014).

Model calibration was done by tuning the maximum recruitment parameter $R_{\max,i}$ for the three fish species while holding temperatures at $T_{\mathrm{ref}}$. $R_{\max,i}$ determines the maximum number of offspring that can be produced by a population in a given time step and serves as a density independent cap on reproduction. This parameter determines how species will respond to exploitation and perturbations, and is one of the main parameters that is calibrated in multi‐species models (e.g., Blanchard et al., 2014; Jacobsen et al., 2017). We used the “L‐BFGS‐B” algorithm (Byrd et al., 1995) in the ‘R’‐optimization function ‘*optim*’ to minimize the residual sum of squares between the natural log of spawning stock biomass estimated in stock assessment output (ICES, 2013, 2015) and those emergent in the model for the years 1992–2002. The optimization procedure resulted in close agreement between SSB from the model and from stock assessments in the calibration time frame. Projections from 1992 and 2012 also generally tracked the assessment SSBs (correlation coefficients of 0.65, 0.94 and 0.54 for cod, herring and sprat, respectively). However, hindcasts (1974–2012) revealed no correlation between assessment SSB and the model, while for herring and cod, the general trends were captured relatively well. The low ability to reconstruct historical biomasses is likely due to the regime shift occurring between 1988–1993 (Möllmann et al., 2009). Growth curves emerging from the model were in close agreement with von Bertalanffy curves fitted to length‐at‐age data from trawl surveys (Figure S6), after a stepwise manual increase of the constant in the allometric maximum‐consumption rate ($h_{i}$) (Appendix S1). The level of density dependence imposed by the stock‐recruitment function (see Equations 14 and 15) was also evaluated by assessing the ratio of the physiological recruitment, $R_{\mathrm{phy},i}$, to the recruitment $R_{i}$ (Jacobsen et al., 2017; Appendix S1). These final values mean that stock recruitment is sensitive to the stock biomass, but there is some density dependence limiting recruitment (i.e., not all spawn produced become recruits). The fishing mortality leading to the highest long‐term yield ($F_{\mathrm{MSY}}$) from the model (estimated for one species at the time while keeping each species at their mean assessment $F_{\mathrm{MSY}}$) were in agreement with the assessment $F_{\mathrm{MSY}}$ for sprat and herring. For cod, $F_{\mathrm{MSY}}$ is lower in the size‐spectrum model than in stock assessments.

### 2.5 Analysis of responses to warming

We analyse the effects of warming on the size‐structure food web in two different ways: by projecting the food web to 2050 with time‐varying sea surface trends, and by projecting the model for 200 years with fixed temperatures above or below $T_{\mathrm{ref}}$. The first set of simulations aimed to assess possible fish population responses to the expected temperature changes, while the second was aimed at exploring effects of temperature on fisheries yield and $F_{\mathrm{MSY}}$ at steady state.

For the time‐varying simulations, models were projected with historical annual fishing mortalities (1974–2012; ICES, 2013, 2015) and sea surface temperature trends (1970–2050, acquired from the regional coupled model system RCA4‐NEMO under the RCP 8.5 scenario; Dieterich et al., 2019; Gröger et al., 2019). These relative temperature trends (relative to mean in 1970–1999) are scaled by adding a constant so that the average temperature in the in the calibration time period is $T_{\mathrm{ref}}$ (10°C). To ensure steady state was reached before time‐varying fishing mortality and temperature was introduced (1974 and 1970, respectively), we applied a 100‐year burn‐in period using the first fishing mortality and temperature value in the respective time series (Figure S12). For each species, we used the fishing mortality at maximum long‐term (‘sustainable’) yield, $F_{\mathrm{MSY}}$ in the years 2012–2050 (Figure S12). These were derived from the size spectrum model by finding the fishing mortalities resulting in highest yields at $T_{\mathrm{ref}}$ (Figure S9). We evaluated the effects of warming on weight‐at‐age, population mean weight and abundance‐at‐weight for each species. This was done for both absolute values, and by comparing warming food webs in 2050 to a baseline scenario where no warming occurred post 1997 (the mid‐point of calibration time window, where temperature averages $T_{\mathrm{ref}}$; Figure S12). In this way, the three scenarios considered contrast the effects of temperature affecting fish physiology, their resources or both.

For the non‐time varying temperature projections we specified a range of constant (not time‐varying) temperatures and fishing mortalities, expressed as proportions of $T_{\mathrm{ref}}$ and $F_{\mathrm{MSY}}$ at the reference temperature ($F_{\mathrm{MSY},T_{\mathrm{ref}}}$), respectively, and projected the models to steady state (200 years). We explored scenarios were temperature ranged between 0.75 to 1.25 of $T_{\mathrm{ref}}$, and $F_{\mathrm{MSY}}$ ranged between 0.1 and 2 of $F_{\mathrm{MSY},T_{\mathrm{ref}}}$. With the full factorial combination of these scenarios, this gave a total of 1989 scenarios. These simulations were done to explore the effect of temperature on fisheries yield and $F_{\mathrm{MSY}}$.

## 3 RESULTS

### 3.1 Effects of warming on size‐at‐age depend on physiological temperature‐dependence

**FIGURE 2:**
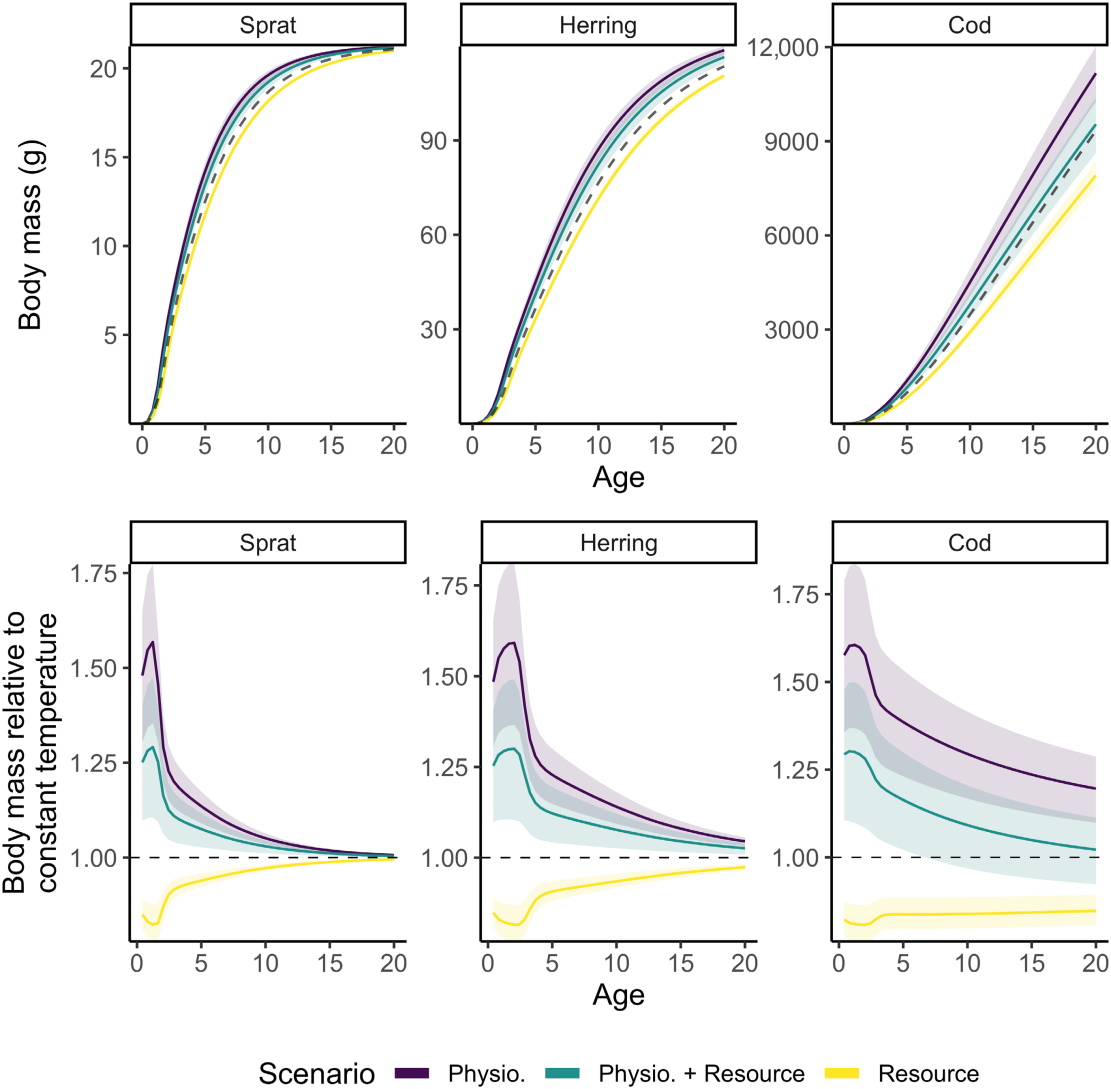
Individual growth trajectories of sprat, herring and cod from model projections to year 2050 assuming warming according to RCP 8.5 while keeping fishing mortality at *F*_MSY_ levels from the size spectrum model. Top row shows size‐at‐age and bottom row shows size‐at‐age relative to a non‐warming scenario. The dashed line in the top row depicts projections assuming a non‐warming scenario and thus constitutes a baseline prediction. Colours indicate different temperature‐scaling scenarios. Shaded areas encompass the 2.5 and 97.5 percentiles from the set of 200 simulations with randomly assigned activation energies.

The inclusion of temperature effects on fish physiological processes has a strong influence on the projected size‐at‐age in 2050 under the RCP 8.5 emission scenario, relative to the baseline projection (no warming; Figure 2). Temperature‐dependence of feeding rates have a particularly large effect (Figure S15). Warming positively affects size‐at‐age when temperature affected metabolism, maximum consumption, search volume and mortality, regardless of whether temperature impacted background resource dynamics (Figure 2). In contrast, the scenarios without temperature‐dependent physiological processes all lead to size‐at‐age decreasing with warming (Figure 2). In scenarios with temperature‐dependent physiological processes, the effects on size‐at‐age are positive and declines with age. When only resources are affected by temperature, small individuals have the largest relative decrease in size‐at‐age, and this negative effect of warming declines with age (Figure 2).

Despite the relatively narrow range of activation energies for physiological rates considered here (Figure S3; Table S3), the uncertainty in projected size‐at‐age associated with variation in the activation energies is large (Figure 2). In the scenario where both physiology and resources are affected by temperature, the range of predicted changes in size‐at‐age vary at approximately +10% to +40% (Figure 2). These changes in size‐at‐age seem to be driven by the temperature‐dependence of maximum consumption rate $\left(h_{i}w^{n}\left(T\right)\right)$ increasing the actual consumption rates $\left(f_{i}\left(w\right)h_{i}w^{n}\left(T\right)\right)$, but having almost no effect on the feeding or satiation levels (Equation 6; Figure S13).

### 3.2 Fewer large individuals cause reductions in mean population body size

**FIGURE 3:**
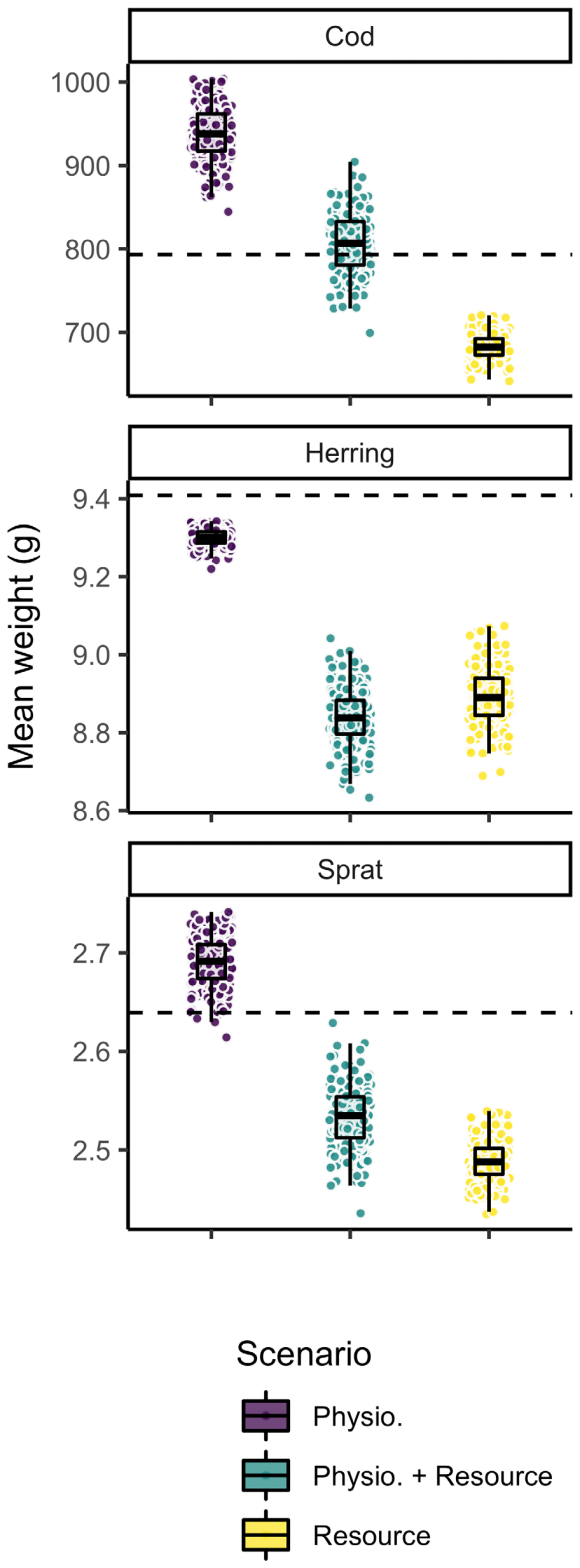
Mean weight across all individuals in the populations of sprat, herring and cod from model projections to year 2050 assuming warming according to RCP 8.5 while keeping fishing mortality at *F*_MSY_ levels from the size spectrum model. The dashed horizontal line depicts projections assuming no temperature increase and thus constitutes a baseline prediction. Each dot represents one of the 200 simulations, each with randomly assigned activation energies. Boxplots depict 25%, 50% and 75% quantiles of the 200 simulations in each scenario.

**FIGURE 4:**
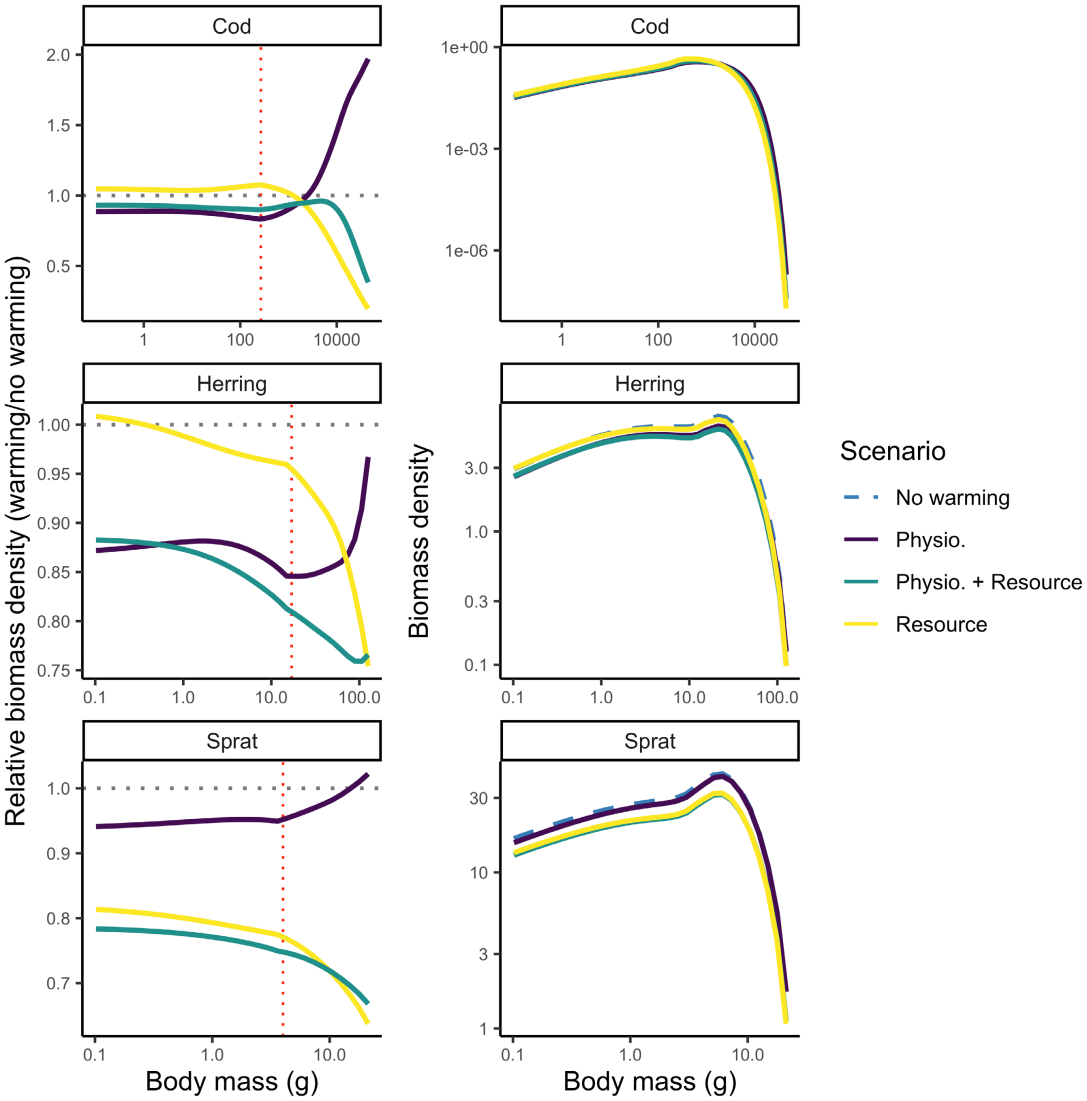
Projected abundance‐at‐weight by species for different scenarios of temperature scaling indicated by colours (and line types in the right column due to overplotting) in 2050 assuming fishing mortality held at *F*_MSY_ levels from the size spectrum model. The left column shows abundance‐at‐weight relative to a non‐warming scenario and the right column shows absolute abundance‐at‐weight with the non‐warming scenario shown in black. Vertical red dotted line indicates weight‐at‐maturation and horizontal black dotted lines indicate the baseline projection (no warming). Only mean activation energies are used (Table S3).

Increases in size‐at‐age (Figure 2) do not always lead to increased mean body size in the populations (Figure 3), due to changes in the relative abundances at size and in this way shifting population size structure (Figure 4). Changes in the size‐structure varied across species without a clear and consistent pattern across species and scenarios.

The only scenario where mean body weight on average increases is where temperature only affects physiology and not the resource (Figure 3). In such cases, body weight increases with warming, but only for cod and sprat. For cod this increase is strong and is driven by both faster growth rates (larger size‐at‐age) and large increases in the abundance of large fish (~10 kg; Figures 2 and 4). For sprat the mean body weight in the populations increased only marginally and is mostly driven by faster growth rates and increased relative abundance of fish above 10 g (Figures 2 and 4). In contrast, scenarios where only resources are affected by temperature, relative numbers of large individuals and therefore mean body size of all species goes down. For herring, all scenarios lead to smaller mean body sizes in the population, and the relative (to non‐warming simulation) abundance‐at‐weight declines with mass in most of the size range, with increases only in the very smallest size classes (<1 g; Figure 4).

### 3.3 Temperature and fishing: Higher sustained exploitation rates but reduced yields in warmer environments

**FIGURE 5:**
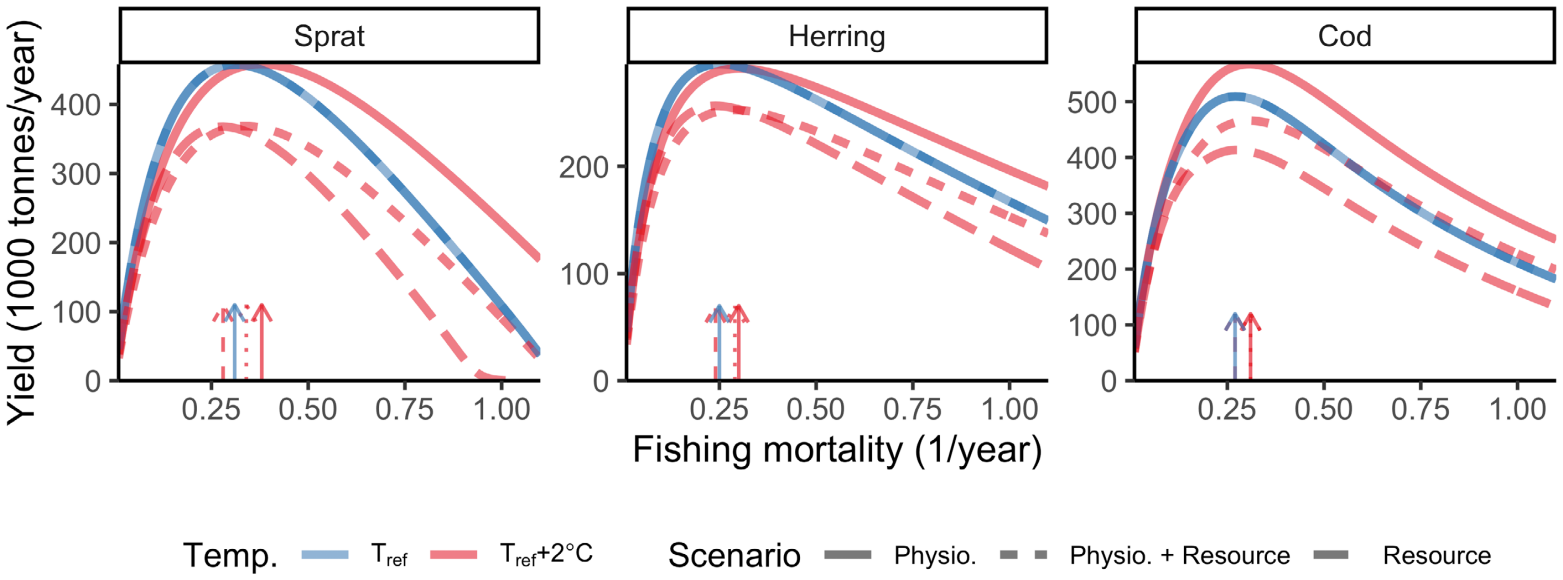
Steady state biomass yield assuming knife edge selectivity at maturation size under two constant temperature simulations and three scenarios for temperature dependence. Colours indicate temperature, where blue means $T=T_{\mathrm{ref}}$ (i.e., no temperature effects), and red depicts warm temperature, here $T=T_{\mathrm{ref}}+2{}^{\circ}\;C$. dashed lines correspond to resource dynamics being temperature dependent, dotted lines correspond to physiological rates and resource dynamics being temperature dependent and solid lines depicts only physiological temperature scaling. Arrows indicate fishing mortality (*F*) that leads to maximum sustainable yield ( $F_{\mathrm{MSY}})$. *F* is held constant at the mean *F* during calibration (mean 1992–2002) for the two other species while estimating yield curves for one species. Note the different scales between species. Only mean activation energies are used (Table S3).

**FIGURE 6:**
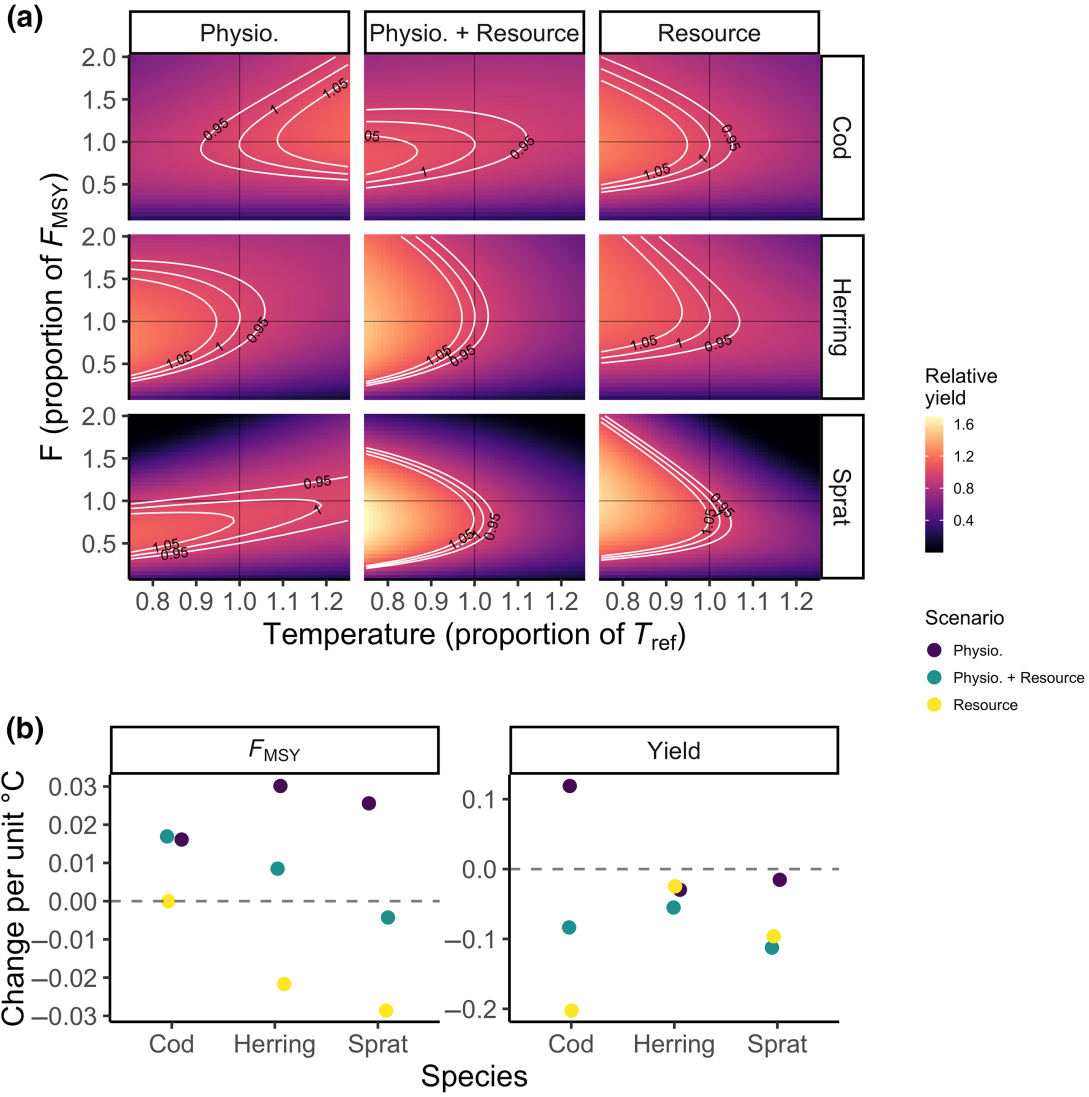
Example of fisheries yield (a) at steady state relative to MSY at $T_{\mathrm{ref}}$ (no effect of temperature) from simulations with constant (not time‐varying) temperatures with the three temperature dependence scenarios (columns), and how $F_{\mathrm{MSY}}$ and yields change with temperature. In panel a, the *y*‐axis shows fishing mortality, F, as a proportion to $F_{\mathrm{MSY}}$ (as estimated from the size spectrum model) at $T_{\mathrm{ref}}$ and the *x*‐axis shows temperature as a proportion of $T_{\mathrm{ref}}$. The other two species are held at their $F_{\mathrm{MSY}}$ when one species' *F* is varied. Panel b shows slopes of $F_{\mathrm{MSY}}$ (left) and yield (right) over temperature. Only mean activation energies are used (Table S3).

Our simulations applying a range of stable (not time‐varying) temperature and fishing scenarios showed that warming led to higher or equal $F_{\mathrm{MSY}}$ (i.e., the fishing mortality leading to maximum sustainable yield), but lower yields (Figures 5 and 6). $F_{\mathrm{MSY}}$ declines with warming for herring when only resources are temperature dependent, and $F_{\mathrm{MSY}}$ for sprat declines resources are temperature dependent, else $F_{\mathrm{MSY}}$ increases. Yields however, decline for all species in all scenarios except for cod when only physiological processes are temperature dependent. The increase in $F_{\mathrm{MSY}}$ is likely due to the enhanced growth rates, which allow higher fishing mortalities without impairing population growth. Cod in the scenario with only physiological scaling is the exemption. The model projects higher yields as temperatures increase, due to the increase in growth rate, average size and relative abundance of large individuals (see Figures 2 and 4). In general, the highest relative yield is found at the coolest temperatures and F slightly lower than $F_{\mathrm{MSY}}$ at the reference temperature (Figure 6). The decline in relative yields of herring and sprat in all scenarios (Figure 5) is likely driven by the warming‐induced decline in abundance, due to resource limitation (Figure 4).

## 4 DISCUSSION

### 4.1 Combined temperature impacts on fish growth rates, body size and fisheries yield

Using a size‐structured and species‐resolved food web model, we demonstrate how climate warming affects growth rates (size‐at‐age), population mean size and size‐structure of interacting exploited fish species and assess its implications for fisheries yield. To do so, we contrasted the effects of warming on resource productivity and individual level physiology (metabolism, feeding and background mortality) of fish. We found that warming leads to increased size‐at‐age of fishes when temperature‐dependence is included in physiological rates. This effect is strongest in juveniles of all three fish species. Though, despite increased growth rates, in most cases, warming leads to smaller mean body size in the population, lower spawning stock biomass (biomass of mature fish) and reduced yields. When temperature affects only the background resource species, the size‐at‐age declines for fish of all sizes.

Mechanistic models that explore warming‐driven declines in community‐wide average body size often find these declines to be driven by lower food abundance or decreased energy transfer efficiency in the food web, due to a combination of declines in plankton density and shifts towards dominance of smaller plankton at higher temperatures (Lefort et al., 2015; Woodworth‐Jefcoats et al., 2015, 2019). This leads to a community wide decline in mean size of fish, where large bodied species become relatively fewer. The cause of these community‐level changes are different from those expected at an individual species level, where temperature can either lead to size‐at‐age changes over ontogeny (in accordance with the TSR), or a change in the relative abundance of small versus large individuals. TSR (the temperature‐size rule) predicts higher growth rates and thus size‐at‐age of juveniles, but smaller adult body sizes (Atkinson, 1994), although the physiological processes that lead to these changes remain debated (Audzijonyte et al., 2019). In our model, we include scenarios that reflect both warmer temperatures impact on food abundance as well physiological changes in metabolism and food intake rates. Scenarios with only temperature dependence of resource dynamics lead to declines in size‐at‐age (that in addition were strongest in young fish). This does not match general observations and predictions of how body growth is affected by warming (Huss et al., 2019; Lindmark et al., 2022; Morita et al., 2010; Thresher et al., 2007), and is not in accordance with the TSR. In contrast, inclusion of physiological temperature dependence leads to projections more in line with general observations from field data, which often find increased size‐at‐age that is strongest and positive for small individuals, and that this effect diminishes over ontogeny (Huss et al., 2019; Thresher et al., 2007).

The increase in body growth that we in find is in general not sufficient for maintaining similar mean population body sizes and size‐structure, if resource carrying capacities decline with warming, because this reduces the relative abundance of large fish. Mean body size in the population and yields therefore decline in the scenario with temperature dependence of both resource dynamics and physiology. These predictions on the net effect of warming are in line with similar models using empirically derived static plankton spectra (Blanchard et al., 2012; Canales et al., 2016; Woodworth‐Jefcoats et al., 2019), and empirical studies (van Dorst et al., 2019). If, however, resource carrying capacity would not decline with temperature, our results show that the increased body growth potential in fish due to higher metabolic and feeding rates can lead to changes towards dominance of larger fish in some populations. This is important to consider, given that predictions about effects of climate change on primary production are uncertain and show large regional variability (Steinacher et al., 2010). These results show that it is important to account for both direct (physiology) and indirect (resources) effects of temperature in order to explain results such as increased growth rates and size‐at‐age but overall smaller‐bodied populations, as also found in other studies (Gårdmark & Huss, 2020; Neubauer & Andersen, 2019; Ohlberger, 2013; Ohlberger et al., 2011).

In fisheries stock assessment, plastic body growth was generally thought to be less important for stock dynamics than environmentally driven recruitment variation, density dependence at early life stages and mortality (Hilborn & Walters, 1992; Lorenzen, 2016). Due to the accumulating evidence of time‐varying and climate‐driven changes in vital rates (survival, growth and reproduction), their relative importance for fisheries reference points and targets are now becoming acknowledged (Lorenzen, 2016; Thorson et al., 2015). In our modelling system, we find that maximum sustainable yields (MSY) and the fishing mortality leading to MSY, that is, $F_{\mathrm{MSY}}$, vary with both temperature and between modelling scenarios, and largely depends on the net effect of temperature on abundance‐at‐size and body growth rates. When temperature affects both the background resources (mainly declining carrying capacity) and fish physiology, warming tends to increase $F_{\mathrm{MSY}}$, but the yield (MSY) derived at this exploitation rate is lower. The decline in yields with warming is due to reduced resource availability, lowering overall fish abundance, and is in line with earlier studies (Blanchard et al., 2012; Lotze et al., 2019). In addition, the warming‐induced decline in relative abundance of fish above minimum size caught in fisheries further decreases yields in our model. At the same time, higher growth rates (size‐at‐age), occurring when temperature affects metabolism and intake rates in particular, can cause $F_{\mathrm{MSY}}$ to increase with warming (Thorson et al., 2015). These reference levels should not be viewed as absolute reference points, and the specific results may depend on the model calibration procedure. However, our findings suggest that climate change predictions on fisheries productivity must consider both temperature impacts on vital rates, in particular body growth, as well as bottom‐up processes and their effects on both the overall abundance and size‐structure of the stock. It also indicates that because productivity may decline with warming in large parts of the oceans (Kwiatkowski et al., 2020; although there is large variation in these predictions across ecosystems [Steinacher et al., 2010]), reduced fisheries yields may be common in a warming world.

### 4.2 Parameterizing and modelling temperature effects

Including physiological temperature‐dependence can strongly influence predictions of warming‐effects and it allows for detailed understanding of temperature effects on populations and food webs via both individual bioenergetics and the emerging responses in fish body growth rates. However, it also requires more parameters, which in turn may vary across species. This could reduce generality of predictions and increase challenges in parameterizing models of data poor systems. We approached this by applying random parameterization, rather than fixed values of temperature dependence. To capture the uncertainty of our approach, we sampled parameters from distributions based on estimates of activation energies of physiological rates in the literature (Lindmark et al., 2022). This approach revealed that in terms of body growth and mean body size in populations, the combination of activation energies can determine whether the mean size increases or decreases with warming, and at what age body sizes decline relative to the current temperatures (degree of decline in size‐at‐age). Hence, better knowledge of the temperature‐dependence of rates of biological processes is needed and these parameters should be chosen carefully, and their uncertainty acknowledged in future modelling studies.

To disentangle temperature effects on background resources and physiological processes, we modelled temperature dependence of resources by scaling their parameters with the same general Arrhenius equation (Gillooly et al., 2001) that we used to scale the physiological processes in fish. Other similar studies that use size spectrum models with physiological temperature‐dependence instead import the plankton spectra from climate and earth systems models (Woodworth‐Jefcoats et al., 2019) or from satellite data (Canales et al., 2016). Such approaches may lead to predictions that are more relevant for a specific system. However, it also becomes more difficult to separate the mechanisms behind the observed changes, as the resource dynamics then are externally forced and cannot respond to changes in the modelled food web. Moreover, populating a resource size spectrum based on observed data can be difficult as observed spectra result from both predation and bottom‐up processes. As an alternative, our approach of directly scaling the carrying capacity or turnover rates of background resources with temperature provides a coherent way to model temperature‐dependencies across trophic levels. The resource dynamics are then impacted by any warming‐driven changes in predators, as well as inherent temperature‐dependent dynamics, rather than driven by external data (Canales et al., 2016) or models (e.g., Woodworth‐Jefcoats et al., 2019). On the downside, this approach means relying on many major simplifications with respect to resource dynamics. In addition, our scenarios only include identical temperature dependencies and baseline carrying capacity of pelagic and benthic resources, and only negative effects of temperature on resource carrying capacity. These may reflect the global decline in primary production (Steinacher et al., 2010) commonly predicted by coupled climate models. It would be straightforward to model increases in carrying capacity with our approach by using positive activation energies. It is also possible to include temperature‐effects of the slope of the size spectrum, as this is often found to be negatively related to temperature (e.g., Canales et al., 2016; Morán et al., 2010; Woodworth‐Jefcoats et al., 2019; Yvon‐Durocher et al., 2011; but see also Barnes et al., 2011 for a non‐significant negative effect on the size‐spectrum slope).

## 5 CONCLUSION

Ecological forecasting is inherently difficult, and climate change alters the already complex causal pathways that drive ecosystem dynamics. Size spectrum models have successfully been used to evaluate size‐based mechanisms and structuring forces in ecosystems (Andersen & Pedersen, 2009; Reum et al., 2019; Szuwalski et al., 2017). In this study, we have highlighted the important role of temperature‐dependent individual‐level metabolism and feeding rates for emerging size‐at‐age patterns that are in line with general observations and predictions (e.g., with the TSR). These also affect the levels of exploitation that leads to maximum sustainable yields, and the corresponding yields. Hence, accounting for temperature‐dependence of both ecological and physiological processes underlying population dynamics is important for increasing our understanding of how and by which processes climate change affects individuals in food webs and resulting effects on fisheries yields, which is needed to generalize across systems and into novel conditions.

## AUTHOR CONTRIBUTIONS

The code was first developed from *mizer* (Scott et al., 2014) by Asta Audzijonyte to include multiple background resources, all authors contributed to developing the code to include temperature. Max Lindmark conceived the idea. All authors contributed to study design. Max Lindmark parameterized the model with input from Anna Gårdmark. Max Lindmark performed analysis and wrote the first draft. All authors contributed to writing the paper and to revisions.

## CONFLICTS OF INTEREST

The authors have declared that no conflict of interests exist.

## Supporting information


Appendix S1

## Data Availability

All model code (parameterization, calibration and analysis) and data are available on GitHub (https://github.com/maxlindmark/mizer‐rewiring/tree/rewire‐temp) and have been deposited on Zenodo (https://doi.org/10.5281/zenodo.6821926).
